# Rapid Molecular Identification of Human Taeniid Cestodes by Pyrosequencing Approach

**DOI:** 10.1371/journal.pone.0100611

**Published:** 2014-06-19

**Authors:** Tongjit Thanchomnang, Chairat Tantrawatpan, Pewpan M. Intapan, Oranuch Sanpool, Penchom Janwan, Viraphong Lulitanond, Somjintana Tourtip, Hiroshi Yamasaki, Wanchai Maleewong

**Affiliations:** 1 Research and Diagnostic Center for Emerging Infectious Diseases, Khon Kaen University, Khon Kaen, Thailand; 2 Faculty of Medicine, Mahasarakham University, Mahasarakham, Thailand; 3 Division of Cell Biology, Department of Preclinical Sciences, Faculty of Medicine, Thammasat University, Rangsit Campus, Pathum Thani, Thailand; 4 Department of Parasitology, Faculty of Medicine, Khon Kaen University, Khon Kaen, Thailand; 5 Department of Medical Technology, School of Allied Health Sciences and Public Health, Walailak University, Thasala, Nakhon Si Thammarat, Thailand; 6 Department of Microbiology, Faculty of Medicine, Khon Kaen University, Khon Kaen, Thailand; 7 Department of Parasitology, National Institute of Infectious Diseases, Tokyo, Japan; AC Camargo Cancer Hospital, Brazil

## Abstract

*Taenia saginata, T. solium*, and *T. asiatica* are causative agents of taeniasis in humans. The difficulty of morphological identification of human taeniids can lead to misdiagnosis or confusion. To overcome this problem, several molecular methods have been developed, but use of these tends to be time-consuming. Here, a rapid and high-throughput pyrosequencing approach was developed for the identification of three human taeniids originating from various countries. Primers targeting the mitochondrial cytochrome *c* oxidase subunit 1 (*cox1*) gene of the three *Taenia* species were designed. Variations in a 26-nucleotide target region were used for identification. The reproducibility and accuracy of the pyrosequencing technology was confirmed by Sanger sequencing. This technique will be a valuable tool to distinguish between sympatric human taeniids that occur in Thailand, Asia and Pacific countries. This method could potentially be used for the molecular identification of the taeniid species that might be associated with suspicious cysts and lesions, or cyst residues in humans or livestock at the slaughterhouse.

## Introduction

Human taeniasis is a disease caused by several *Taenia* spp., including *Taenia saginata, T. solium*, and *T. asiatica* [1]–[3]. Taeniids have a worldwide distribution [4]. Their definitive hosts are carnivores that become infected after eating larvae (cysticerci) contained in meat from herbivorous intermediate hosts. The larvae develop to adulthood in the small intestine and shed gravid proglottids, which pass to the outside in feces and disintegrate to release the eggs contained within. Eggs ingested by grazing herbivores develop to cysticerci in muscles and elsewhere. Human taeniasis is acquired from inadequately cooked infected pork (*T. solium* and *T. asiatica*) or beef (*T. saginata*). For many years there was debate about the status of *T*. *asiatica*, with many scientists considering it to be a variant of the beef tapeworm because of similarities in the adult worms [5]. It was named as a new species in 1993 [2]. Amongst the features distinguishing it from the beef tapeworm are morphological details of the cysticerci and the site where these are found (mostly the liver of pigs, whereas cysticerci of *T*. *saginata* are found mostly in muscles of bovids).

In the case of *T. solium*, if humans ingest eggs, these can develop into cysticerci in organs such as the brain, causing cysticercosis [1]. Cysticercosis, especially neurocysticercosis, due to *T*. *solium* can be fatal in humans [1]. *Taenia saginata* and probably *T*. *asiatica* are relatively harmless in humans: the adult worms occur in the small intestine and cause only a few specific symptoms, such as abdominal pain and nausea [6]. While *T. solium* and *T. saginata* have worldwide distributions, *T. asiatica* [5] is found mostly in Asian countries, including Korea, China, Taiwan, Thailand, Indonesia, Vietnam, Philippines, and Japan [7], [8]. The early detection and adequate treatment of taeniasis is important for the prevention of *T. solium* infection and/or cysticercosis [9]. Necessary epidemiological tools for effective control of taeniasis or cysticercosis include accurate methods for parasite identification. At present, the morphological identification of proglottids and scolices expelled from tapeworm carriers, or of cysticerci collected from intermediate hosts, are the routine approaches that are employed.

In areas where these cestodes are sympatric, distinguishing between proglottids of *T. saginata* and *T. asiatica* is impeded by their strong morphological similarities. The diagnosis of taeniasis by microcoscopy using fecal samples from patients is unreliable because the eggs of these species are very similar. The only reliable and completely accurate approach for the differentiation of taeniid eggs, cysticerci and proglottids is the use of DNA-based methods. Several PCR tests/assays targeting various genomic regions are available for differentiating human *Taenia* infections. These include PCR-restriction fragment length polymorphism (RFLP) methods [10]–[11], loop-mediated isothermal amplification (LAMP) [12], the use of species-specific DNA probes [13], the BESS T-base method [14], PCR for the differentiation of *T. saginata* and *T. solium* [15], multiplex PCR for the detection of *T. saginata* and *T. asiatica* and of the American/African and Asian genotypes of *T. solium* using primers from the *cox1* gene [16], a high-resolution multiplex PCR assay [17] and a nested PCR system [18]. Analyzing the results from these methods requires gel electrophoresis, which is slow, has a limited throughput, and is prone to carry-over contamination and subjective results.

A rapid DNA sequencing method, pyrosequencing, is a promising tool for yielding diagnostic data. The method relies on determining the sequence of addition of nucleotides to a primer complementary to a single stranded template. As a nucleotide is incorporated into the growing complementary strand by a polymerase, pyrophosphate is released which is converted to ATP by ATP sulphurylase. The ATP in turn is an energy source for luciferase, allowing it to oxidise luciferin and generate a detectable quantity of light [19]. Light is only generated when a newly added nucleotide is complementary to the next unpaired base in the template strand. The intensity of light is proportional to the number of sequential identical bases in homopolymers, but determining the exact length of a homopolymer can be a problem for this technique [19]. Pyrosequencing has been used in a broad range of applications, such as SNP genotyping, *de novo* mutation detection, gene identification and microbial genotyping [20]. It has recently been developed for the species-level identification of *Entamoeba* species [21] and has been applied to several parasite taxa, such as *Plasmodium* [22], *Paragonimus* [23] and *Trichinella* species [24]. The use of pyrosequencing for the identification of human taeniids has not yet been reported. The present study aimed to develop a pyrosequencing method for sequencing amplicons from *cox1* for the identification of *Taenia* species that are parasites of humans and intermediate hosts.

## Materials and Methods

### Parasite and DNA Materials

Forty-seven specimens of expelled proglottids were collected from patients who visited hospitals in Thailand, Japan, and China for medical treatment (Table 1). One cysticercus specimen was collected from a dead pig in the Kathmandu market, Kathmandu, Nepal; one cysticercus specimen was collected from a dead pig in the Cocal dos Alves market in Cocal dos Alves City, Piracuruca, Piaui State, northeastern Brazil; and one cysticercus specimen was from beef in the Saitama meat inspection center, Saitama City, Saitama Prefecture, Japan. All Thai samples of *T. saginata* (Table 1) were collected from patients who visited the Srinagarind Hospital, Faculty of Medicine, Khon Kaen University, Khon Kaen, Thailand. Additional *T. saginata* samples and two samples of *T. solium* proglottids were from patients of various nationalities treated in hospitals in Tokyo, Japan. A *Taenia asiatica* proglottid from China (case 8) was from a Chinese patient who was treated in a hospital in Dali, Yunnan province. In this case, the proglottid was expelled during mass treatment in the township. Three further *T. asiatica* samples were from Japanese patients who were treated in hospitals in the Tokyo area. The human cases diagnosed in Japan have been previously described [8]. In every case, each worm was analyzed individually.

**Table 1:** Characteristics and molecular identification of the 47 taeniasis cases examined in this study.

Patient				scolex/proglottidsexpelled	Locality	SangerSequencing	Pyrosequencing
Case No.	Sex	Age, y	Year				
1	M	30	2007	proglottid	Thailand	*T. saginata*	*T. saginata*
2	M	36	2007	proglottid	Thailand	*T. saginata*	*T. saginata*
3	M	65	2007	proglottid	Thailand	*T. saginata*	*T. saginata*
4	M	76	2007	proglottid	Thailand	*T. saginata*	*T. saginata*
5	M	48	2008	proglottid	Thailand	*T. saginata*	*T. saginata*
6	M	59	2008	proglottid	Thailand	*T. saginata*	*T. saginata*
7	F	61	2008	proglottid	Thailand	*T. saginata*	*T. saginata*
8	NR	NR	2008	proglottid	China	*T. asiatica*	*T. asiatica*
9	M	50	2009	proglottid	Thailand	*T. saginata*	*T. saginata*
10	M	56	2009	proglottid	Thailand	*T. saginata*	*T. saginata*
11	M	63	2009	proglottid	Thailand	*T. saginata*	*T. saginata*
12	M	76	2009	proglottid	Thailand	*T. saginata*	*T. saginata*
13	F	23	2009	proglottid	Thailand	*T. saginata*	*T. saginata*
14	F	67	2009	proglottid	Thailand	*T. saginata*	*T. saginata*
15	F	68	2009	proglottid	Thailand	*T. saginata*	*T. saginata*
16	F	NR	2009	proglottid	Thailand	*T. saginata*	*T. saginata*
17	NR	NR	2009	proglottid	Thailand	*T. saginata*	*T. saginata*
18	F	20	2009	proglottid	India*	*T. solium*	*T. solium*
19	M	19	2010	proglottid	Thailand	*T. saginata*	*T. saginata*
20	F	37	2010	proglottid	Thailand*	*T. saginata*	*T. saginata*
21	M	31	2010	proglottid	India*	*T. solium*	*T. solium*
22	M	58	2010	proglottid	Japan*	*T. asiatica*	*T. asiatica*
23	M	40	2010	proglottid	Japan*	*T. asiatica*	*T. asiatica*
24	F	39	2010	proglottid	Japan*	*T. asiatica*	*T. asiatica*
25	M	25	2011	proglottid	Thailand	*T. saginata*	*T. saginata*
26	M	28	2011	proglottid	Thailand	*T. saginata*	*T. saginata*
27	M	51	2011	proglottid	Thailand	*T. saginata*	*T. saginata*
28	M	88	2011	proglottid	Thailand	*T. saginata*	*T. saginata*
29	M	NR	2011	proglottid	Thailand	*T. saginata*	*T. saginata*
30	F	51	2011	proglottid	Thailand	*T. saginata*	*T. saginata*
31	F	70	2011	proglottid	Thailand	*T. saginata*	*T. saginata*
32	M	33	2011	proglottid	Sudan*	*T. saginata*	*T. saginata*
33	F	24	2011	proglottid	Ethiopia*	*T. saginata*	*T. saginata*
34	F	42	2011	proglottid	France*	*T. saginata*	*T. saginata*
35	M	21	2012	proglottid	Thailand	*T. saginata*	*T. saginata*
36	M	28	2012	proglottid	Thailand	*T. saginata*	*T. saginata*
37	M	50	2012	scolex and proglottid	Thailand	*T. saginata*	*T. saginata*
38	M	50	2012	proglottid	Thailand	*T. saginata*	*T. saginata*
39	M	56	2012	proglottid	Laos	*T. saginata*	*T. saginata*
40	M	59	2012	proglottid	Thailand	*T. saginata*	*T. saginata*
41	M	62	2012	proglottid	Thailand	*T. saginata*	*T. saginata*
42	M	70	2012	proglottid	Thailand	*T. saginata*	*T. saginata*
43	M	75	2012	proglottid	Thailand	*T. saginata*	*T. saginata*
44	M	78	2012	proglottid	Thailand	*T. saginata*	*T. saginata*
45	M	60	2012	proglottid	Mali*	*T. saginata*	*T. saginata*
46	M	89	2013	proglottid	Thailand	*T. saginata*	*T. saginata*
47	F	51	2013	proglottid	Thailand	*T. saginata*	*T. saginata*

NR, no record.

*The samples were from patients who were treated in hospitals in Tokyo, Japan.

All of the procedures used in this study were approved by the Human Ethics Committee of Khon Kaen University, based on the Ethics of Human Experimentation of the National Research Council of Thailand (Reference No. HE561396) and ethical approval (No. 177) of the Ethics Committee of the National Institute of Infectious Diseases, Tokyo, Japan. Because the parasites were recovered from diagnostic specimens acquired during routine laboratory investigations, and from fecal specimens from patients who visited the hospital for medical treatment, documented informed consent was not required by the Ethics Committees. Patient anonymity was maintained. The study was made up of research on pre-existing specimens. Specimens of cysticerci that were obtained from local markets and slaughterhouses do not correspond to the PLoS criteria for observational and field studies, and therefore separate ethics clearance was not required for these. No vertebrate specimens were used.

### Primer Design

**Figure 1:**
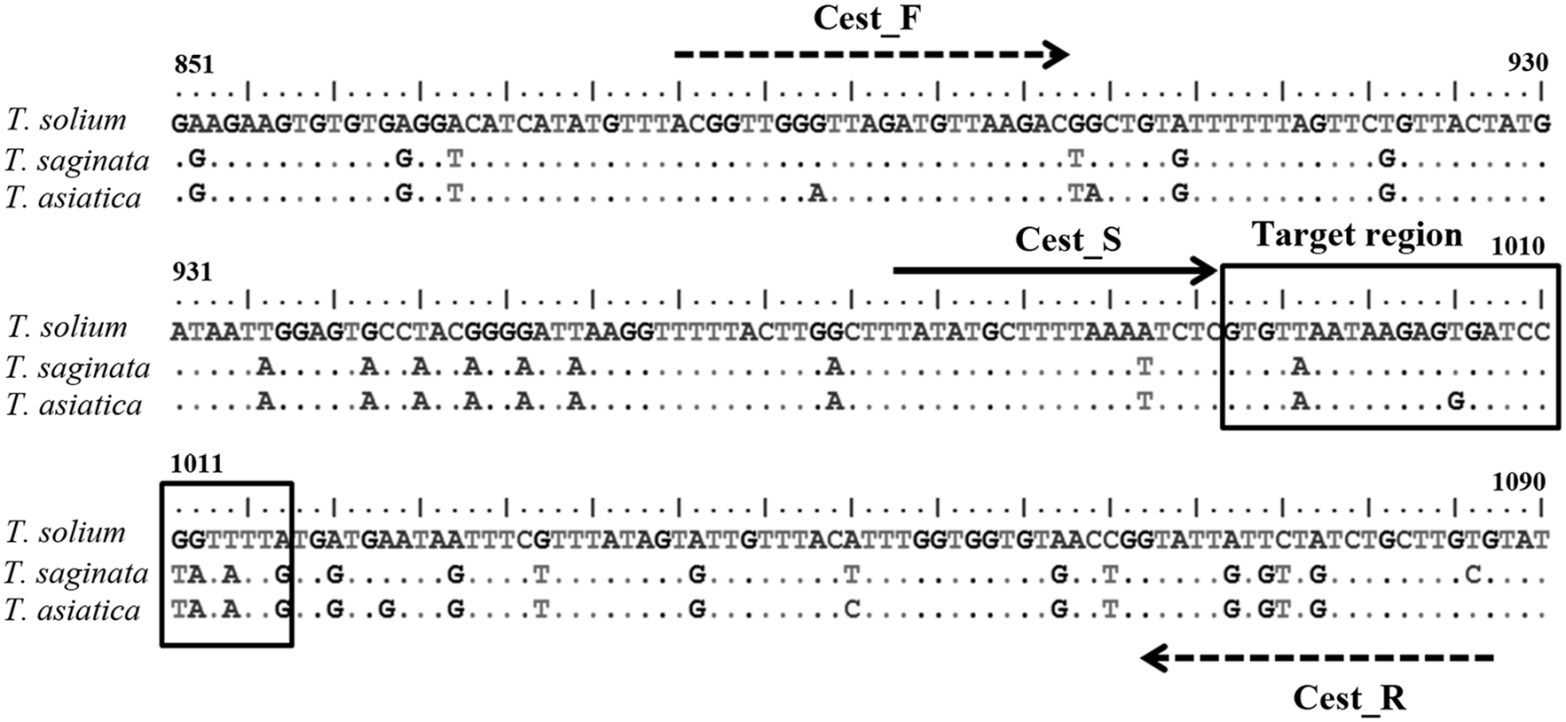
Alignment of the mitochondrial *cox1* gene and design of primers. Alignment of the mitochondrial *cox1* gene derived from *T. solium* (AB524785), *T. saginata* (AB645845) and *T. asiatica* (AB608739). The broken arrows indicate the position of Cest_F (forward primer) and the biotinylated Cest_R (reverse primer) for template amplification. The solid arrow indicates Cest_S (sequencing primer), and the rectangular box shows the position (992–1017) of the target region used for species level identification. The dots indicate identical nucleotides between the sequences.

Sequences of the *cox1* gene (1,620 bp) of *T. solium* (GenBank accession no. AB524785), *T. saginata* (AB645845) and *T. asiatica* (AB608739) were used to guide primer design (Fig. 1). Based on these data, PCR primers (Cest_F; 5′-ACGGTTGGGTTAGATGTTAAGAC -3′, corresponding to positions 880–902 and biotinylated Cest_R; biotin-5′- CGCAAGCAGACAACACAATAC - 3′, corresponding to positions 1087–1067) were designed. A sequencing primer, Cest_S (5′- TATATGCTTTTAAATTCTC -3′, corresponding to positions 973–991), was also designed using pyrosequencing assay design software (PyroMark Q96 ID software version 2.0; Biotage, Uppsala, Sweden) (Fig. 1). Sequences generated from this primer will differ between the three taeniid species (Fig. 1).

### DNA Extraction and Plasmid Preparation

DNA was extracted from an individual proglottid of *T*. *saginata* obtained from each patient in Khon Kaen, Thailand, using the Nucleospin Tissue kit (Macherey-Nagel GmbH & Co, Duren, Germany). The DNA was eluted in 50 µl of distilled water, and 2 µl of the sample was used for each PCR reaction. The DNA samples were stored in a DNA bank at −70°C in the Department of Parasitology, Faculty of Medicine, Khon Kaen University until used. In all remaining cases, DNA was extracted from each sample using the DNeasy Blood & Tissue kit (Qiagen, Hilden, Germany). For evaluation of the specificity, DNA samples from other parasites were used, including *Ascaris lumbricoides*, hookworm, *Trichuris trichiura*, *Capillaria philippinensis*, *Strongyloides stercoralis*, *Trichostrongylus* spp., *Schistosoma mekongi*, *Schistosoma japonicum*, *Opisthorchis viverrini*, *Haplorchis taichui*, *Clonorchis sinensis*, *Centrocestus* spp., *Stellantchasmus* spp., *Paragonimus heterotremus*, *Fasciola gigantica*, *Echinostoma malayanum*, intestinal lecithodendriid flukes, *Giardia duodenalis* and *Isospora belli.* Genomic DNA samples from human leukocytes (blood sample donated by W.M., approved by the Human Ethics Committee of Khon Kaen University) and beef and pork from dead animals (from the Khon Kaen market, Khon Kaen, Thailand) not infected with any taeniids were also analyzed.

Positive control plasmids of each of the *Taenia* species were constructed by ligation of each species-specific amplicon into a pGEM-T easy vector (Promega, WI), according to the manufacturer’s instructions. Plasmids were propagated in *Escherichia coli* JM109 cells. Each inserted gene was sequenced in both directions using the Sanger method, and the resulting sequences were identical to the gene sequences from which the primers had been designed.

### DNA Amplification by Polymerase Chain Reaction (PCR) and Pyrosequencing

The *cox1* gene fragments (208 bp) were amplified from *Taenia* DNA samples. Each PCR reaction had a final volume of 25 µl containing 1×PCR buffer (Invitrogen, Carlsbad, CA) with 0.2 mM of each dNTP, 2 mM MgSO_4_, 0.2 µM of Cest_F primer, 0.2 µM of biotinylated Cest_R primer, 0.625 U of Platinum *Taq* DNA polymerase high fidelity (Invitrogen) and 2 µl of the DNA template. The amplification procedure was as follows: 5 min at 94°C for the initial denaturation, 35 cycles of denaturation for 30 s at 95°C, annealing for 30 s at 52°C, and extension for 30 s at 72°C, followed by a final extension for 10 min at 72°C. The molecular sizes of the amplicons were verified by electrophoresis on 1.5% agarose gels. After the PCR amplification, the biotinylated PCR products were placed in 96-well plates containing 0.4 µM Cest_S sequencing primer and processed by using the PyroMark Q96 ID instrument (Biotage) as previously described [22], [23]. Following the completion of the pyrosequencing reaction, a pyrogram was produced and analyzed. The readouts were interpreted manually in case the target sequences contained homopolymers longer than four bases.

## Results

**Figure 2:**
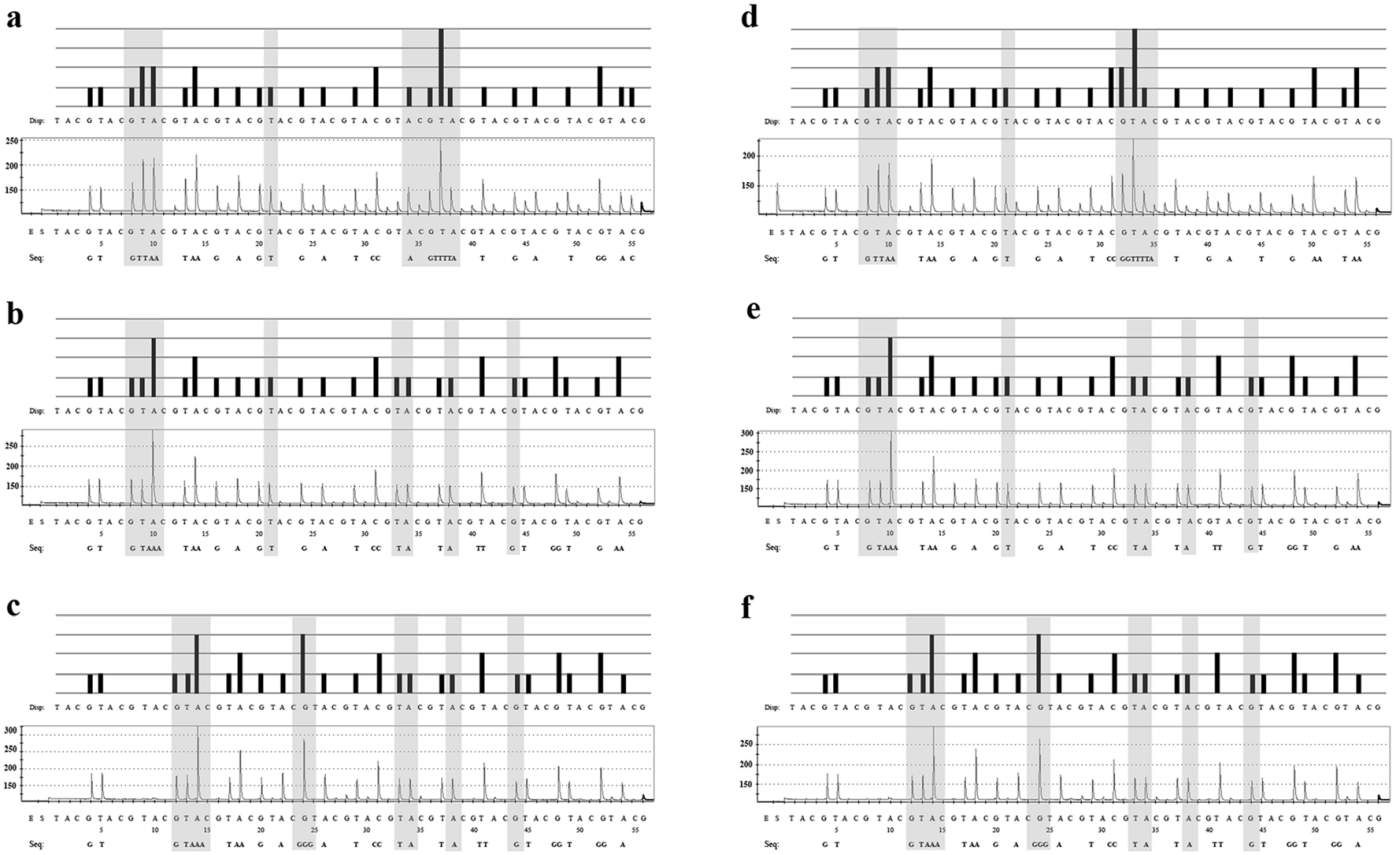
Sequence analysis pyrograms of *T. solium T. saginata* and *T. asiatica cox1* genes. Pyrograms showing the sequence analysis of 26-base fragments of the cox1 gene of T. solium (a, d) T. saginata (b, e) and T. asiatica (c, f) using pyrosequencing. The theoretical pyrogram patterns (top of each panel) and representative raw data from the control plasmids (a, b, c) and DNA extracted from taeniid proglottids (d, e, f) from pyrosequencing (bottom of each panel) are shown. The pyrosequencing was performed by the addition of enzyme (E), substrate (S), and four different nucleotides dispensed sequentially. The letters under the black bars show the dispensation (Disp:) order. The actual sequence detected by the pyrosequencing is indicated below the panels after “Seq:”. The Y-axis represents the level of fluorescence emitted by the incorporation of a nucleotide base, and the X-axis represents the total number of bases added at that point in time; A, C, G, T, nucleotide bases. The light gray bars show the regions that can be used to distinguish between T. solium, T. saginata and T. asiatica.

The 26 nucleotides adjacent to the 3′ end of the sequencing primer were sufficient as a target region to distinguish between the three *Taenia* species using the pyrosequencing technique (Fig. 1, Fig. 2, Table 2). All of the results showed the expected sequence (Fig. 1, Table 2). *Taenia solium* could be discriminated from *T. saginata* and *T. asiatica* by nucleotides at 5 positions, and *T. asiatica* was distinct from *T*. *saginata* at one nucleotide position (Table 2). The reproducibility of the pyrosequencing approach was confirmed by Sanger sequencing of the amplicons, which yielded identical sequence data. The specificity of the PCR primer set was analyzed, and the results showed that no amplification product was detected with the DNA of other parasites or genomic DNA from human leukocytes or uninfected beef and pork (data not shown).

**Table 2:** Nucleotide patterns of the *cox1* gene to differentiate *Taenia solium, T. saginata* and *T. asiatica* using the pyrosequencing technique.

Species (Accession No.)	Nucleotide at positions					
	996	1005	1011	1012	1014	1017
***T. solium*** (AB524785, AF360866)	T^a^	T	G/A^a^	G^a^	T^a^	A^a^
***T. saginata*** (AB645845)	A	T	T	A	A	G
***T. asiatica*** ** (**AB608739**)**	A	G^b^	T	A	A	G

a Specific nucleotide to distinguish *T. solium* from *T. saginata* and *T. asiatica.*

b Specific nucleotide to distinguish *T. asiatica* from *T. solium* and *T. saginata.*

## Discussion

*Taenia solium*, *T. saginata* and *T. asiatica* are sympatric in a number of Asian countries. Differentiating between *T. saginata* and *T. asiatica* proglottids is difficult because of their morphological similarities. It is hard to differentiate between the eggs of all three species using morphological features. A rapid, reliable and high-throughput molecular method to differentiate between these taeniid cestodes is therefore important for epidemiological and clinical reasons [7], [8], [16], as outlined in the introduction.

Previous molecular approaches to species identification/diagnosis i.e. the PCR-RFLP technique [10] the base excision sequence scanning thymine-base method [14], the multiplex PCR [16], the nested-PCR method [18], and the LAMP method [12], [25], [26], reviewed in the introduction, all have drawbacks. Most require many pairs and primers and gel or polyacrylamide electrophoresis; this electrophoresis is sluggish, has a limited throughput and is prone to carry-over contamination. The pyrosequencing technique is such a method. All of the steps, from the DNA extraction to pyrosequencing, can be completed within 4 h with the same degree of accuracy as Sanger sequencing, but without the need for labeled nucleotides, labeled primers and electrophoresis. Using pyrosequencing, a large number of samples can be analyzed in a short time, and at much lower cost for reagents [27]. Some limitations of this approach include the short read lengths (25–50 bp) and that long homopolymers, if present, create problems for the analysis. The latter problem can be resolved by manual evaluation of the pyrograms. In our experiments, pyrosequencing yielded exactly the same data as Sanger sequencing of PCR amplicons for the 26 nucleotides of the target region. The pyrosequencing technique can be used as an effective means for the identification of *T. saginata, T. asiatica* and *T. solium.* The primer set used in this present study was highly specific for *Taenia* species and did not amplify products from other parasites or genomic DNA from human leukocytes or uninfected beef and pork. A future application could be to use this technique for the species-specific identification of taeniid eggs or DNA in fecal samples, allowing for the identification of *T. solium*, *T. saginata* and *T. asiatica* without the need for proglottid samples. This identification application is important for the treatment and prevention of *T. solium,* which, being a causative agent of neurocysticercosis, is the most serious taeniid species for humans. More *T. asiatica* isolates should be investigated in future work to determine whether there is any intra-specific variation in the target region, especially in countries from which we did not have samples. Another potential use is the identification of tapeworm species suspected of producing cysts or other lesions in livestock at the slaughterhouse. The application of the technique needs to be further evaluated with those parasites/pathogens that might be misidentified by visual inspection.

We have presented here a rapid and reliable approach to distinguishing between the main species of *Taenia* that infect humans. This basic method can be modified to apply to other species of cestode, or indeed of any pathogens. The pyrosequencing method also lends itself to high-throughput analyses that might be required for broad-scale surveys.
